# HEALTH STATUS OF OLDER VIETNAMESE REFUGEES: RESULTS FROM THE VIETNAMESE AGING AND CARE SURVEY (VACS)

**DOI:** 10.1093/geroni/igz038.1376

**Published:** 2019-11-08

**Authors:** Christina E Miyawaki, Nai-Wei Chen, Oanh L Meyer, Mindy Thy Tran, Kyriakos S Markides

**Affiliations:** 1 Graduate College of Social Work, University of Houston, Houston, Texas, United States; 2 Beaumont Research Institute, Beaumont Health, Royal Oak, Michigan, United States; 3 University of California, Davis, Sacramento, California, United States; 4 University of Houston, Houston, Texas, United States; 5 University of Texas Medical Branch, Galveston, Texas, United States

## Abstract

Over 1.3 million Vietnamese including refugees migrated to the U.S., after the Vietnam War. Vietnamese are the 4th largest Asian ethnic group in the U.S. Despite the number, little is known about their health conditions. To fill this gap, the Vietnamese Aging and Care Survey (VACS) was developed, and sociodemographic and health data on 132 refugees (≥65 years) were collected in Houston, Texas. They were on average 75.4 years-old, retired (77%), married (58%), female (55%) with less than high school education (86%) in poor/fair health (76%). They immigrated around age 49 years-old, and have hypertension (74%), arthritis (48%), and diabetes (41%). They manage their lives by living in a multi-generation tightly-knit enclaves, and show resilience to their low sociodemographic status (≤25K, 94%). Findings suggest healthcare professionals to introduce more social services such as adult daycare programs in culturally-sensitive ways to ease their transition to new lives in the U.S.

